# Integrating Non-technical Skills in Endoscopy into Gastroenterology Training: A National Framework for Saudi Arabia

**DOI:** 10.7759/cureus.112158

**Published:** 2026-07-06

**Authors:** Diamond Joy, Syed Anjum Gardezi, Nassir Hayaf, Eman Al Sulais, Turki Al-Ameel

**Affiliations:** 1 Gastroenterology and Hepatology, Johns Hopkins Health System, Johns Hopkins Aramco Healthcare, Dhahran, SAU; 2 Gastroenterology and Hepatology, Johns Hopkins Health System, Dhahran, SAU; 3 Gastroenterology and Hepatology, Johns Hopkins Aramco Healthcare, Dhahran, SAU; 4 Internal Medicine, Royal Commission Hospital, Jubail, SAU; 5 Internal Medicine, King Fahad Specialist Hospital, Dammam, SAU

**Keywords:** communication, education, endoscopy, gastroenterology training, interpersonal and communication skills, kingdom of saudi arabia (ksa), medical education, non-technical skills, saudi arabia

## Abstract

The practice of gastroenterology has evolved rapidly over the past two decades, driven by advances in endoscopic technology, expanding therapeutic indications, and increasing expectations of patient safety and quality of care. While technical competence in endoscopy remains a cornerstone of gastroenterology training, there is growing recognition that non-technical skills in endoscopy (NTSE) are also critical determinants of procedural success and patient outcomes. NTSE encompasses cognitive, interpersonal, and behavioral competencies, including communication, situational awareness, decision-making, leadership, teamwork, professionalism, and ethical practice within the endoscopy unit. However, their incorporation into gastroenterology training remains variable, particularly in the Middle East.

In Saudi Arabia, gastroenterology training programs have made substantial progress in standardizing technical endoscopic training, yet formal curricula addressing non-technical skills are limited. This narrative review examines the rationale for integrating NTSE into gastroenterology training programs in Saudi Arabia, reviews relevant international evidence, and proposes a contextually appropriate framework for curriculum development, implementation, and assessment. Embedding non-technical skills training within existing gastroenterology curricula will enhance patient safety, improve trainee performance, and align Saudi training programs with international best practices. We outline practical strategies for faculty development, assessment methods, and future research priorities to support sustainable implementation.

## Introduction and background

Gastroenterology is a procedure-intensive specialty in which endoscopy plays a central diagnostic and therapeutic role. Although technical competence is routinely assessed through procedural metrics and certification pathways, non-technical skills (NTS) such as communication, teamwork, situational awareness, and decision-making are less consistently taught and assessed despite their recognized importance for patient safety and procedural performance. This gap has prompted increasing international interest in structured non-technical skills training within endoscopy. Increasing evidence suggests that adverse events, inefficient procedures, and patient dissatisfaction frequently arise not just from technical failure but also from deficiencies in non-technical skills such as communication, teamwork, situational awareness, and decision-making [1,2].

Non-technical skills represent cognitive and behavioral competencies that enable clinicians to function effectively in complex clinical environments. In endoscopy, these competencies, collectively termed non-technical skills in endoscopy (NTSE), support safe procedural performance, coordinated teamwork, and appropriate clinical judgment during dynamic procedural situations [1,2].

Healthcare disciplines, such as surgery and anesthesia, have already incorporated structured frameworks for training in non-technical skills [3-6]. These approaches have been associated with improvements in team performance, crisis management, and patient safety. Similar principles apply to endoscopy, where procedures are performed in time-pressured environments requiring coordination between physicians, nurses, technicians, and trainees [2].

Despite growing international recognition of NTSE, structured integration into gastroenterology training remains inconsistent in many regions [1]. In Saudi Arabia, gastroenterology training programs have achieved substantial progress through the Saudi Commission for Health Specialties (SCFHS) Adult Gastroenterology Curriculum, which adopts the CanMEDS competency framework [7]. However, behavioral competencies related to communication, teamwork, and decision-making are typically acquired informally through apprenticeship rather than structured curriculum design [1].

Despite the incorporation of CanMEDS competencies within the SCFHS curriculum, there is limited published evidence describing structured NTSE teaching, assessment, or documentation within Saudi gastroenterology training programs. This narrative review outlines the conceptual foundations of NTSE, summarizes current evidence supporting non-technical skills training in endoscopy, examines the status of NTSE within Saudi gastroenterology training, and proposes a practical national framework for integrating these competencies into fellowship programs. This article proposes a roadmap for integrating non-technical skills into gastroenterology training in Saudi Arabia.

Literature selection

Relevant literature was identified through targeted searches of PubMed, Google Scholar, and reference lists of key articles using terms related to non-technical skills, endoscopy training, human factors, simulation, assessment tools, and Saudi gastroenterology training. The selection prioritized peer-reviewed studies, consensus statements, validation studies, and training frameworks most relevant to the development of a practical NTSE integration roadmap for Saudi Arabia. This article represents a narrative review and expert perspective. Given the heterogeneity of the available evidence, including educational frameworks, assessment tools, validation studies, and training models, no formal quantitative synthesis or meta-analysis was performed.

Background and conceptual framework

Non-technical skills refer to the cognitive, social, and personal resource skills that complement technical expertise and contribute to safe clinical performance. In endoscopy, these competencies include communication, teamwork, situational awareness, leadership, decision-making, professionalism, and the ability to manage stress during procedures.

The concept of non-technical skills (NTS) originated from the aviation industry, where human factors were identified as major contributors to accidents. These insights led to the development of crew resource management (CRM) [8], which emphasized structured communication, shared situational awareness, and team coordination to improve operational safety. NTS has been incorporated into healthcare, where complex tasks are performed under time pressure, resulting in fewer errors and better clinical outcomes [3,9,10].

One influential framework is Non-Technical Skills for Surgeons (NOTSS), which categorizes performance across domains including situation awareness, decision-making, communication and teamwork, and leadership [4]. Similarly, Anesthetists' Non-Technical Skills (ANTS) was developed for improving practice [6].

Building on these human-factors principles, the United Kingdom Joint Advisory Group (JAG) on Gastrointestinal Endoscopy developed the Endoscopic Non-Technical Skills (ENTS) behavioral marker system [2]. This framework adapts NOTSS concepts to the endoscopy environment and assesses domains such as communication, teamwork, situational awareness, leadership, decision-making, and coping with pressure.

NTSE has been incorporated into the United Kingdom training pathway through the JAG Endoscopy Training System (JETS) e-portfolio, wherein trainees receive structured feedback on behavioral performance on endoscopy procedures [11,12]. The integration of NTSE into certification processes demonstrates that non-technical skills can be explicitly taught, observed, and assessed, providing a model for competency-based endoscopy education. The key domains of NTS are described below.

__PRESENT

__PRESENT__PRESENT__PRESENT

__PRESENT

## Review

Communication

Effective communication is a cornerstone of NTSE and underpins patient safety, procedural efficiency, and team performance. Clear, timely, and structured verbal communication with nursing staff and anesthesia providers is essential before and during procedures, particularly during critical moments such as sedation escalation or therapeutic interventions [1]. Non-verbal communication, including situational cues and body language, plays an important role in a noisy and time-pressured endoscopy environment. Communication with patients and families before the procedure should ensure informed consent, realistic expectations, and understanding of potential risks and outcomes. Post-procedural communication is equally important for conveying findings, follow-up plans, and warning signs. Breakdowns in communication are a common contributor to adverse events and near-misses in endoscopy units [1]. Developing deliberate communication strategies, such as closed-loop communication and structured briefings, enhances overall procedural safety and quality.

Teamwork and collaboration

Strengthening teamwork through training, briefings, and debriefings is, therefore, central to safe and effective non-technical skills [13]. NTSE emphasizes the ability to function effectively within a multidisciplinary endoscopy team. High-quality endoscopic care depends on coordinated interactions between endoscopists, nurses, technicians, anesthesia providers, and trainees. Understanding and respecting each team member's roles and responsibilities improves workflow efficiency and reduces errors. Effective collaboration fosters shared situational awareness and allows early identification of patient deterioration or procedural challenges. A culture of mutual respect encourages team members to speak up when concerns arise, regardless of hierarchy. Poor teamwork has been linked to delays in complication recognition and suboptimal responses to emergencies.

Situational awareness

Situational awareness refers to the ability to perceive, comprehend, and anticipate changes in the clinical environment during endoscopy. This includes continuous assessment of patient vital signs, sedation depth, procedural progress, and equipment function [1]. Loss of situational awareness may occur due to cognitive overload, fixation on the endoscopic image, or environmental distractions. Maintaining situational awareness allows the endoscopist to recognize early signs of complications such as hypoxia, bleeding, or perforation. It also supports timely adjustments in technique or procedural strategy. High-level situational awareness is particularly critical during complex or prolonged procedures. In NTSE, this skill differentiates technically competent endoscopists from those who consistently deliver safe and reliable care [14].

Decision-making and judgment

Sound clinical judgment is a defining element of NTSE in gastrointestinal endoscopy. Endoscopists must integrate patient factors, procedural findings, risk profiles, and available resources to make timely and appropriate decisions. This includes deciding when to proceed, modify technique, escalate care, or abort a procedure in the interest of patient safety [1]. Decision-making under uncertainty is common in endoscopy, particularly during therapeutic interventions or unexpected findings. Cognitive biases, time pressure, and fatigue can negatively influence judgment if not recognized. Effective NTSE involves reflective practice and awareness of personal limitations. Developing structured decision-making frameworks and learning from adverse events can improve judgment and outcomes.

Leadership and followership

Leadership in NTSE involves guiding the endoscopy team toward shared goals while maintaining patient safety and procedural efficiency [2]. The endoscopist often assumes a leadership role, particularly during complex procedures or emergencies. Effective leaders provide clear direction, set priorities, and create an environment where team members feel comfortable voicing concerns. Equally important is followership, which involves recognizing when to seek help, defer to others with greater expertise, or accept input from team members. Adaptive leadership allows roles to shift depending on the clinical situation. Poor leadership can contribute to confusion, delays, and increased risk during critical events. Balanced leadership and followership are therefore essential components of high-quality NTSE skill.

Professionalism and ethics

Professionalism and ethical practice are integral to NTSE and extend beyond technical performance. Endoscopists must uphold patient dignity, privacy, and confidentiality throughout the procedural pathway [1]. Ethical practice includes obtaining informed consent, managing conflicts of interest, and ensuring transparency when complications occur [15,16]. Respectful behavior toward patients and team members promotes trust and a positive safety culture. Accountability for clinical decisions and outcomes is a core professional responsibility. Ethical challenges may arise in areas such as appropriateness of procedures, trainee involvement, and disclosure of adverse events. A strong foundation in professionalism ensures that NTSE aligns with both clinical excellence and societal expectations.

Stress and fatigue management

**Figure 1:**
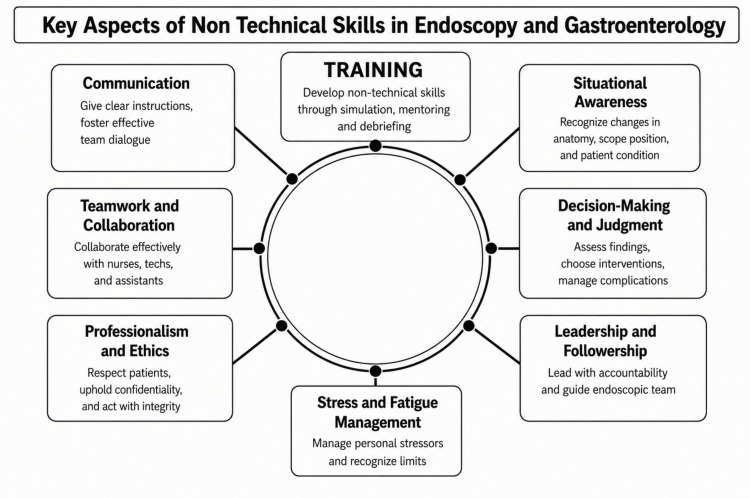
Non-technical skills description.

Managing stress and fatigue is a critical but often under-recognized aspect of NTSE. Endoscopy lists can be physically and cognitively demanding, particularly in high-volume or emergency settings. Excessive stress and fatigue impair attention, decision-making, and situational awareness, increasing the risk of error [1]. Recognizing personal limits and early signs of cognitive overload is essential for maintaining performance. Strategies, such as adequate rest, task prioritization, and seeking support when needed, help mitigate these risks. Organizational factors, including scheduling and workload, also influence stress levels and should be addressed at a systems level. Effective stress and fatigue management supports sustainable, safe, and high-quality endoscopic practice. Table 1 shows how the various NTSEs are used. The key aspects of the non-technical skills are described in Figure 1.

**Table 1:** Examples of non-technical skills across the endoscopy workflow.

Skill	Before procedure	During procedure	After procedure
Communication	Explain indications, risks, and consent	Provide clear instructions to team	Communicate findings and follow-up
Teamwork	Establish shared plan with team	Coordinate actions during procedure	Debrief and review workflow
Situational awareness	Review patient risk factors	Monitor vitals and procedural progress	Identify risk of delayed complications
Decision-making	Assess procedural appropriateness	Modify or stop procedure if needed	Plan further investigation or therapy
Leadership	Assign team roles	Direct team during critical events	Lead reflection and follow-up
Professionalism	Respect autonomy and dignity	Maintain professional conduct	Document and disclose outcomes
Stress management	Ensure readiness and preparation	Manage cognitive load	Reflect on fatigue and performance

Evidence and assessment tools

Growing evidence shows that non-technical skills influence endoscopic performance, team dynamics, and patient safety by shaping communication, recognition of complications, and responses to unexpected events. Various assessment tools have been validated and used to evaluate competence in gastroscopy [17,18], colonoscopy [19,20], polypectomy [21], endoscopic retrograde cholangiopancreatography (ERCP) [22,23], and endoscopic ultrasound (EUS) [24]. These assessment tools focus not just on technical skills, but also on non-technical skills (Table 2). 

NTSEs are increasingly recognized as essential components of safe and effective procedures [1]. Apart from technical skills, the lack of NTS has been found to cause increased errors, complications, and adverse outcomes in endoscopy [25]. Also, analysis of data from the National Reporting and Learning system in the United Kingdom has shown that deficiencies in communication and decision-making skills have been linked to procedural failures, poor patient satisfaction, and adverse outcomes [26].

Performance varies even among experienced endoscopists and cannot be fully explained by established determinants such as demographic factors or adenoma detection rate. Differences in non-technical skills, including judgment, communication, teamwork, and leadership, likely contribute to these noted variations [27]. NTS are particularly important in advanced endoscopy due to procedural complexity and higher risks of adverse events. While traditional risk analyses focus on technical factors [28,29], many strategies for preventing complications, such as appropriate patient selection, familiarity with equipment, and early recognition and management of adverse events, depend largely on non-technical skills.

Training focused on non-technical skills in endoscopy (NTSE) has been found to boost endoscopists' performance [2]. Evidence supporting structured NTSE training also includes simulation-based studies and controlled trials. A randomized controlled trial using simulation-based training on NTSE among novice endoscopists has shown significantly improved NTS scores and colonoscopy performance [30].

Several validated tools have been developed to support competency-based assessment of endoscopic performance. The UK Joint Advisory Group (JAG) has incorporated non-technical skills into national endoscopy training and assessment tools, including Direct Observation of Procedural Skills (DOPS) and the JAG Endoscopy Training System (JETS) e-portfolio [2,31]. The American Society of Gastrointestinal Endoscopy Assessment of Competency in Endoscopy (ACE) tool includes some cognitive skills but less behavioral assessment in evaluating endoscopic competence [31]. The Canadian Gastrointestinal Endoscopy Competency Assessment Tool (GiECAT) has demonstrated strong construct validity and high inter-rater reliability, with intraclass correlation coefficients exceeding 0.80 [17]. A study comparing three assessment tools favored the DOPS as more balanced and reliable, but more validation studies are needed [32].

Although Saudi training programs have high procedural volumes, they rank lower in structured NTSE, simulation-based training, and competency-based non-technical assessment. Some of the assessment tools used by prominent organizations worldwide are shown in Table 2.

**Table 2:** Selected assessment tools incorporating non-technical skills in endoscopy. EGD: esophagogastroduodenoscopy; ERCP: endoscopic retrograde cholangiopancreatography.

Tool	Organization	Procedures	Behavioral domains
Direct Observation of Procedural Skills (DOPS) [11]	Joint Advisory Group on Gastrointestinal Endoscopy-United Kingdom (JAG)	Upper and lower GI	Communication, teamwork, professionalism
Direct Observation of Polypectomy Skills (DOPyS) [11]	JAG	Polypectomy	Decision-making, situational awareness
Assessment of Competency in Endoscopy (ACE) [32]	American Society for Gastrointestinal Endoscopy (ASGE)	EGD and colonoscopy	Cognitive skills, judgment
Gastrointestinal Endoscopy Competency Assessment Tool (GiECAT) [17]	Canadian Association of Gastroenterology (CAG)	General endoscopy	Communication, decision-making
Global Assessment of Gastrointestinal Endoscopic Skills (GAGES) [33]	Society of American Gastrointestinal and Endoscopic Surgeon-United States (SAGES)	Upper and lower GI	Teamwork, safety
Endoscopic Non-Technical Skills (ENTS) [31]	JAG	All endoscopic procedures	Communication, leadership, teamwork
Team Endoscopic Non-Technical Skills (TEAM-ENTS) [31]	JAG	Team performance	Coordination and team communication
Biliary Endoscopy Skills Assessment Tool (BESAT) [22]	ASGE	ERCP	Decision-making and situational awareness

Current state of endoscopic non-technical skills (ENTS) in Saudi Arabia

Gastroenterology training in Saudi Arabia is delivered through accredited residency and fellowship programs overseen by the Saudi Commission for Health Specialties (SCFHS). The Adult Gastroenterology Curriculum adopts the CanMEDS competency framework and emphasizes core roles, including communication, professionalism, and collaboration in postgraduate training [7]. The training has become increasingly standardized, with defined expectations for procedural volume, duration of training, and technical competency. Trainees benefit from high case volumes and exposure to diverse gastrointestinal pathology.

Despite these advances, there is limited published evidence regarding the structured development and assessment of NTSE within Saudi gastroenterology training programs. While the SCFHS curriculum incorporates competencies related to communication, professionalism, and collaboration, the routine use of validated behavioral marker systems, dedicated NTSE curricula, and formal non-technical skills assessment tools has not been well described in the Saudi literature. Training and evaluation continue to focus primarily on technical performance and procedural metrics, while behavioral competencies such as communication, teamwork, situational awareness, leadership, and decision-making are not routinely operationalized through behavioral markers, validated assessment tools, or formal logbook documentation.

Recent national survey data have also identified opportunities to strengthen educational experiences, mentorship, and leadership development within gastroenterology training programs. Accordingly, further work is needed to evaluate current NTSE practices and establish standardized approaches to training and assessment [34]. Feedback on behavioral performance was often informal and dependent on individual supervisors rather than standardized assessment frameworks. Importantly, trainees expressed a strong interest in structured NTSE training.

Several contextual factors may contribute to this variability. Endoscopy units frequently operate in high-volume clinical environments with significant service demands, limiting opportunities for structured teaching, debriefing, and reflective learning. Multidisciplinary teams often include professionals from diverse linguistic and cultural backgrounds, which may complicate communication during complex procedures. Hierarchical clinical structures may also discourage junior trainees from speaking up during procedures.

Collectively, these factors suggest that the acquisition of NTSE in Saudi gastroenterology training currently relies largely on apprenticeship rather than structured curriculum design. Integrating validated behavioral frameworks and standardized assessment tools into training programs would therefore represent an important step toward strengthening competency-based endoscopy education.

Proposed framework for integration

Integrating NTSE into gastroenterology training requires a structured and phased approach aligned with national training standards. A practical framework could be implemented over several years to allow pilot testing, faculty development, and gradual national adoption.

Phase 1: Pilot Implementation

Initial implementation could occur in selected high-volume training centers. During this phase, internationally validated frameworks such as the JAG ENTS behavioral marker system may be adapted for local use [31]. Faculty development workshops would prepare trainers to observe behavioral performance and provide structured feedback. Simulation-based sessions could introduce trainees to key NTSE principles, including communication, teamwork, and situational awareness.

Phase 2: Curriculum Integration

Following pilot evaluation, ENTS teaching could be incorporated into fellowship curricula through workshops, case discussions, simulation training, and structured debriefings after complex procedures [31]. Behavioral assessment tools could be incorporated into trainee portfolios alongside technical competency assessments.

Phase 3: National Adoption

Successful pilot implementation could support broader integration across accredited gastroenterology training programs [31]. Incorporation of NTSE assessments into national logbooks would allow standardized documentation of behavioral competencies across training centers.

Phase 4: Evaluation and Continuous Improvement

Ongoing evaluation would assess the impact of NTSE training on trainee performance and procedural outcomes. Metrics may include trainee assessment scores, simulation performance, team communication measures, and patient-reported experience [30]. Continuous review would allow refinement of training methods and alignment with evolving international standards.

Challenges and considerations

Several challenges may affect the implementation of structured NTSE training. Hierarchical clinical cultures may discourage trainees from speaking up during procedures, limiting open communication within teams [35]. Introducing structured briefings, debriefings, and team-based training can help promote psychological safety and collaborative decision-making.

Workload pressures in busy endoscopy units may also limit opportunities for dedicated training [36]. Integrating NTSE teaching within routine clinical activities, such as pre-procedure team briefings or post-procedure reflections, can help minimize disruption while reinforcing behavioral competencies.

Another challenge is limited faculty experience in assessing non-technical skills. Faculty development programs are therefore essential to train supervisors in behavioral observation, feedback techniques, and the use of validated assessment tools.

Addressing these barriers requires institutional support, alignment with national training priorities, and recognition of non-technical skills as core competencies in endoscopic practice.

__PRESENT

__PRESENT

__PRESENT

__PRESENT

__PRESENT

__PRESENT__PRESENT

## Conclusions

Non-technical skills are essential components of safe, effective, and patient-centered endoscopic practice. While Saudi gastroenterology training programs have achieved strong procedural exposure and technical competence, structured development of behavioral competencies remains limited.

International experience demonstrates that non-technical skills can be explicitly taught, observed, and assessed using validated frameworks. Integrating NTSE into national training programs offers an opportunity to enhance trainee competence in communication, teamwork, situational awareness, and decision-making.

A phased implementation strategy incorporating simulation-based education, structured assessment tools, and faculty development could support the integration of NTSE within Saudi gastroenterology training. Such efforts align with national priorities in patient safety and quality improvement while positioning Saudi Arabia to advance competency-based endoscopy education in the region.
